# Large-Scale Distributed Training of Transformers for Chemical Fingerprinting

**DOI:** 10.1021/acs.jcim.2c00715

**Published:** 2022-10-04

**Authors:** Hisham Abdel-Aty, Ian R. Gould

**Affiliations:** Department of Chemistry and Institute of Chemical Biology, Imperial College London, Molecular Sciences Research Hub, Shepherd’s Bush, LondonW12 0BZ, UK

## Abstract

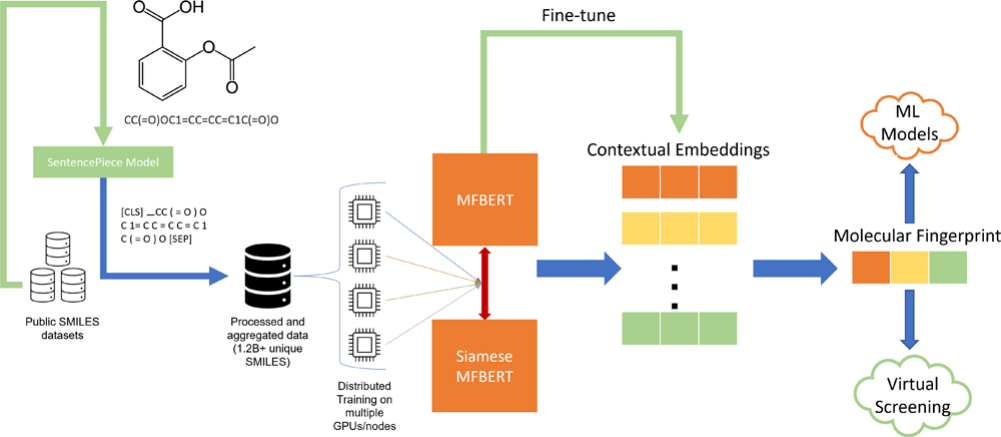

Transformer models have become a popular choice for various machine learning tasks due to their often outstanding performance. Recently, transformers have been used in chemistry for classifying reactions, reaction prediction, physiochemical property prediction, and more. These models require huge amounts of data and localized compute to train effectively. In this work, we demonstrate that these models can successfully be trained for chemical problems in a distributed manner across many computers—a more common scenario for chemistry institutions. We introduce MFBERT: Molecular Fingerprints through Bidirectional Encoder Representations from Transformers. We use distributed computing to pre-train a transformer model on one of the largest aggregate datasets in chemical literature and achieve state-of-the-art scores on a virtual screening benchmark for molecular fingerprints. We then fine-tune our model on smaller, more specific datasets to generate more targeted fingerprints and assess their quality. We utilize a SentencePiece tokenization model, where the whole procedure from raw molecular representation to molecular fingerprints becomes data-driven, with no explicit tokenization rules.

## Introduction

Data-driven chemical prediction techniques have seen a recent surge in performance, usability, and adaptability.^1−6^ The increased size and availability of chemical data and its accessibility means that large machine learning (ML) models can now be trained on these data to achieve better performance on chemical prediction tasks.^6−11^ These models can also learn from unlabeled data through self-supervised training procedures.^12−14^ When combined, these factors contribute to the recent successes of ML techniques for chemical prediction.

Until recently, graph neural networks (GNNs) have been the core model choice for chemical prediction tasks, and deep neural networks have also been used in conjunction with classical molecular fingerprinting algorithms to aid data featurization.^3,5,15,16^ Molecular fingerprinting algorithms such as the extended connectivity fingerprint (ECFC4) use explicit rules to generate a fixed-length vector consisting of extracted molecular features.^17^ These features can then be used as inputs for more complex predictive or generative models. The end-user must determine the fingerprinting algorithm that extracts the features most suited for the downstream task. The quality of each fingerprint can be assessed by observing how they can separate varying classes of molecules in some latent space (for example, in a virtual screening setting) or by comparing the metrics for some downstream prediction task. Classical molecular fingerprints are inflexible since they follow explicitly coded rules and only extract pre-defined molecular features. As such, data-driven fingerprinting approaches have been developed, which can adapt the features extracted based on the training data.^15^ The improvement of predictive and generative models in chemistry is imperative as it allows accelerated progress in drug discovery, catalysis, chemical biology, and other fields at a significantly reduced cost compared to ex silico experiments.

Transformer models^18,19^ and other natural language processing (NLP) techniques are beginning to emerge with the increased availability of chemical data in the text-based simplified molecular input line entry system (SMILES) representation and the development of new training techniques.^20^ Various strategies for training language models on chemical data with various model architectures including transformers, recurrent networks, and autoencoders have been explored. These strategies include devising tasks such as translating a sequence of reactant SMILES to a sequence of product SMILES, using SMILES to predict chemical properties, combining classically computed chemical properties with some other latent representation for property prediction, and using latent representations for reaction classifications.^1,4,21^

Transformer models utilize an attention mechanism to determine the relative importance of each symbol in the tokenized SMILES to every other symbol and itself. Performing this attention computation can generate valuable molecular fingerprints from the output embeddings of the transformer. While SMILES can represent most molecules accurately with some stereochemical information, the limitation of spatial 2D graphs remains. This spatial limitation is more pronounced in the 1D text representation. As such, redundancies in the representation are introduced, whereby one molecule can have multiple valid SMILES representations depending on the molecular graph traversal algorithm. This limitation can be exploited when training large data-driven models on big data such that the SMILES can be augmented to aid the learning of valid structures. However, recent studies have shown that for datasets that are already large, augmenting the data this way provides little added value.^14^

In this work, we evaluate the scaling of a RoBERTa^22^ based architecture, a widely used transformer encoder in NLP, in a distributed manner on chemical data, resulting in the largest transformer model training on chemical data in literature, MFBERT. We quantitively asses our model’s pre-training by measuring the performance of the model on RDKit’s benchmarking platform^23,24^ for virtual screening while offering multiple inference methods for flexibility with fine-tuning on smaller, more targeted datasets with minimal computation.

## Methods

### Data

**Figure 1:**
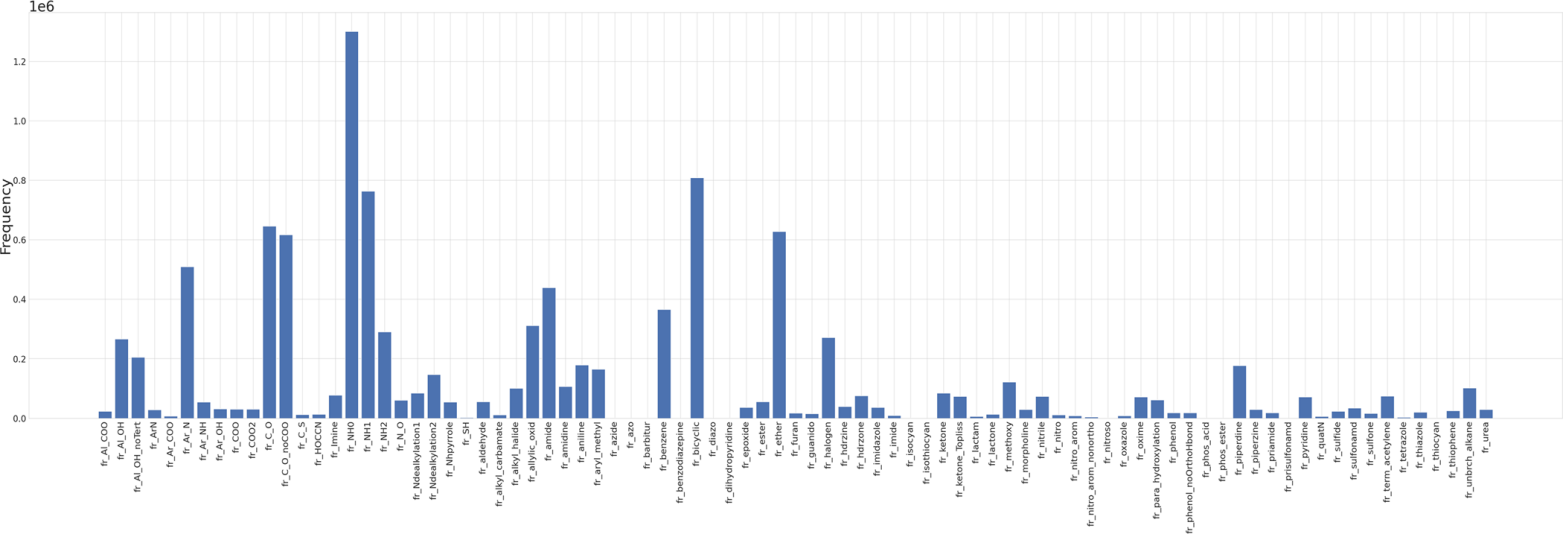
Functional group distribution from a one million SMILES random sample from the aggregate dataset used for pre-training. There are 85 fragments/groups considered according to RDKit’s fragment module.

A selection of publicly available SMILES datasets were aggregated, sanitized, and filtered for pre-training. These datasets were: GDB-13,^8^ Zinc 15,^25^ PubChem,^26^ ChEMBL,^27^ and USPTO.^9^ The relative sizes of the final, filtered datasets are shown in Table 1. For each dataset, the properties of the data were determined and used to generate the overall model pre-training pipeline. GDB-13^8^ is the largest subset proportionally; however, this is a synthetically generated molecular dataset involving the enumeration of all possible molecular graphs with constraints. As such, it served as an initial pre-training dataset to aid model convergence and to allow the model to learn the basic principles of the SMILES representation. The Zinc 15 dataset^25^ consists of catalogs of purchasable organic, drug-like molecules. This dataset was used for training and oversampling to focus the model’s pre-training onto understanding the chemical patterns in real molecules. PubChem^26^ is an open structure database of over 200 M unique chemical structures as of 2020; these structures were also used for larger pre-training with ChEMBL. The ChEMBL dataset^27^ is a collection of hand-selected biologically active molecules. A subset of ChEMBL is used by RDKit’s benchmarking platform for virtual screening;^23,24^ this ChEMBL subset was also used as the validation set for pre-training. For each dataset, we identify, validate, and sanitize unique molecules. We then aggregate the unique identified molecules and perform frequency analysis on the duplicate and non-canonical representations of the unique molecule in the dataset. We then augment the final dataset proportionately according to the frequency analysis. The USPTO dataset^9^ is a reaction dataset consisting of 450k+ reaction SMARTS. Our model was also trained on reaction SMARTS less than 512 tokens long (the model configuration’s limit); this offers flexibility for the tokenizer and for fine-tuning. All molecules were extracted from the USPTO reactions and used for oversampling of common molecules. Each molecule in the aggregate dataset was sanitized and canonicalized using a custom parallel RDKit^28^ Python script. Frequency analysis of the canonical SMILES duplicates from the USPTO reaction molecules was performed. The top 20% most frequent duplicates were used for augmentation in both canonical and non-canonical forms, acting as permutations. In this case, the SMILES permutations were not only used for augmentation, but the frequent molecules were also duplicated proportionately in the pre-training set. In other words, we use SMILES augmentation to introduce common molecules to the model, multiple times in different forms, and we duplicate some SMILES in the training set in proportion to their commonality in reactions. This process was performed on 48 cores for 24 h. All canonical duplicates outside of the frequency threshold (top 20%) were removed. The data was then shuffled using terashuf,^29^ an external-memory shuffling algorithm in linear time, a process that took ∼20 h on eight cores. We take a one million sample subset and compute the functional group distribution across the sample; this is shown in Figure 1.

**Table 1:** Unfiltered and Filtered Pre-Training Dataset Sizes and the Proportions They Represent of the Final Dataset^a^

dataset	GDB-13	Zinc 15	PubChem	ChEMBL	USPTO	total
unfiltered size (molecules)	977,468, 301	389,000,000	206,550,222	1,920,027	994,838	1,575,933,388
proportion of unfiltered total (%)	81	10	9	0.2	0.00825	100

a The filtered aggregate dataset contains only unique SMILES, totaling 1,264,754,823 molecules.

### Tokenization

In the original RoBERTa^22^ implementation, a byte-pair encoding (BPE) tokenizer^30^ was used; this, however, assumes that the text is pre-tokenized (“words” are separated by spaces). As this is not the case with SMILES, in this work, a unigram SentencePiece tokenizer^31^ was used. This tokenizer trains a unigram model^32^ on the dataset and treats the input text as a raw input stream. The generated vocabulary is then dependent on reducing an initial seed vocabulary based on the log-likelihood loss of the unigram model. This custom tokenizer then adds auxiliary tokens such as [CLS] and [SEP] rather than <s > and </s > in the original BPE implementation to denote the start and the end of a token sequence. Previous works have shown that with BERT-like models, the performance on downstream tasks is minimally impacted when using subword tokenizers or regular expressions (RegEx) to treat SMILES as pre-tokenized strings.^12^ We take a one million SMILES random sample from our aggregate dataset and compare the total number of tokens that would be fed through the model (excluding padding). The regular expression that was used in this comparison was
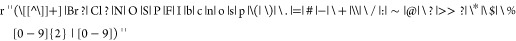


Over 1 million samples, RegEx tokenization results in 2,034,812 extra tokens being fed through the model with, on average, an extra two tokens per SMILES with minimal additional gain on downstream task performance.^12^ Attention is of the order *O*(*n*^2^), where *n* is the number of tokens in the sequence. During inference, SentencePiece offers a more efficient system overall with minimal downsides.

### Attention

**Figure 2:**
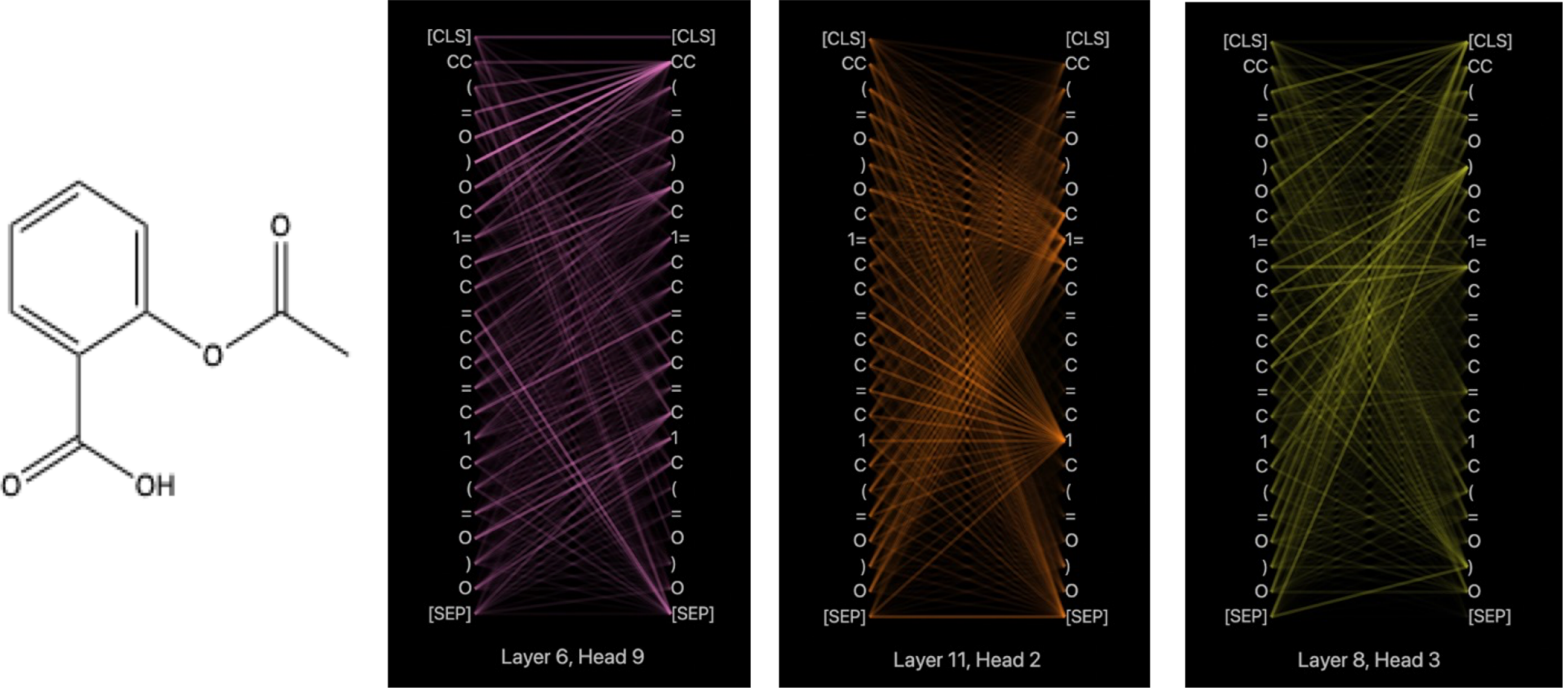
Attention weight visualizations for head 9, layer 6; head 2, layer 11; and head 3, layer 8 of MFBERT on aspirin. Each layer shows the attention mechanism attending to various molecular features within the SMILES. For example, the start and end of the phenyl ring (specifically the “1” and “1=” tokens) are attended to highly by almost all other atoms in layer 11, head 2. In layer 6, head 9, the carbon in the “C=O” of the carbonyl group denoted by token “CC” has a high attention weight to the carbonyl oxygen and its closing branching token “)”.

The premise of transformers relies on there being a sequence of tokens and a task to perform on that sequence. For each token in a sequence, an attention score is computed, relative to every other token and the token itself, based on the weights previously learned during training. These attention weights can offer some level of interpretability for the model; however, due to the large number of attention pairs, it can be difficult to visualize. Figure 2 shows sample attention from layers 6, 8, and 11.

From the attention weights, the SMILES features that the model is attending to can be seen. In this case, for the carbonyl group, the oxygen and its surrounding branching tokens are paying significant attention to the carbonyl carbons in layer 6, head 9, suggesting that the model is somewhat able to recognize and differentiate between coupled atomic groups and their importance to the likelihood of other molecular features around them. This separation is only somewhat representative of the model’s learnings as not all attention heads in each layer seem to learn useful chemical features. From the extracted functional groups, we perform a substructure and pattern matching search to match each functional group to their SMILES tokens and their respective attention weight proportions. We then perform a Mann–Whitney *U* test between the functional group distribution and the distribution of attention weight proportions given to functional tokens. The resultant *p*-value was <0.01. This suggests that although qualitatively, it may appear to be somewhat possible to interpret attention weights, there are other non-interpretable features across the model’s layers and attention heads that carry significant weight in the output fingerprint.

### Model

**Figure 3:**
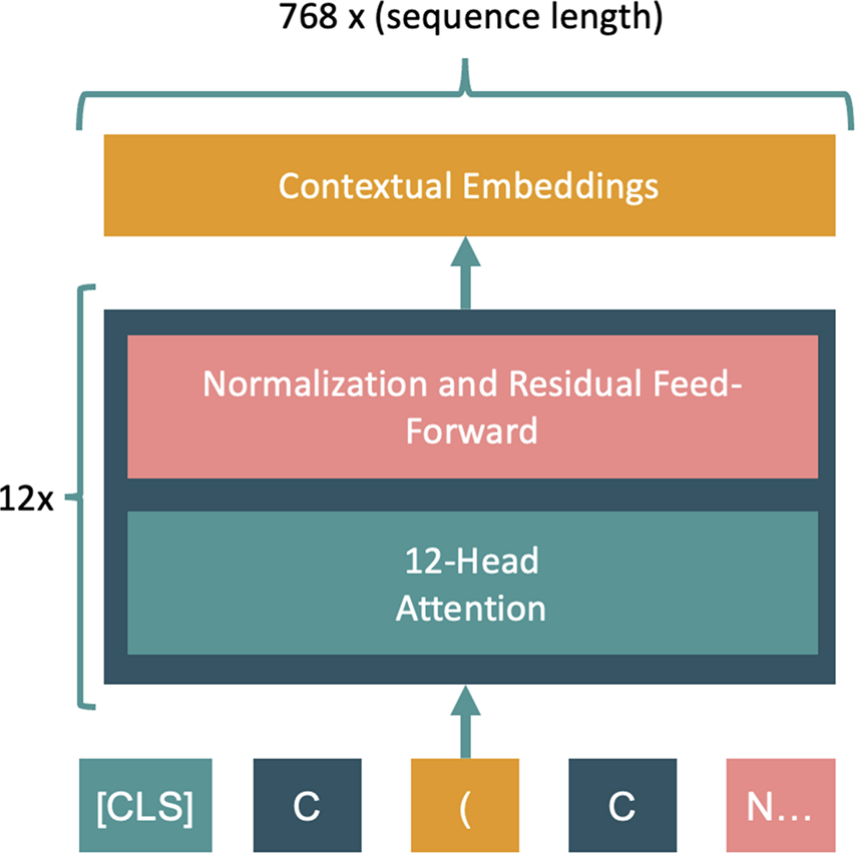
MFBERT architecture. A stacked transformer encoder is fed molecular token embeddings that are attended to by 12 attention heads for 12 blocks. The hidden dimensions are 768, and the max sequence length is truncated to 512 tokens (514 with auxillary tokens). A 768-dimension contextual embedding is given for each input token as output.

The architecture of MFBERT consists of a large stack of transformer encoders. It is based on the RoBERTa^22^ architecture with 12 encoder-attention blocks and 12 attention heads per block. Given a vocabulary size of 2417 tokens, this gives our model a total of ∼88 M trainable parameters. Figure 3 shows the model architecture. Each token is fed through the encoder layers, with each layer returning latent 768-dimensional embeddings for each token. The final layer returns the most accurate representation based on the model’s learning procedure.

### Pre-Training Procedure

For the pre-training task, masked language modeling (MLM)^19^ was used. Fifteen percent of the tokens in the dataset were masked/corrupted, and the model’s task was to uncover the masked tokens. Cross-entropy loss was used as the objective function for this task. For each training stage, a polynomial decay learning rate schedule was used along with a linear increase for warmup. The peak learning rate (LR) used was 0.0006. This was determined to be in proportion to scale the suggested hyperparameters in RoBERTa^22^ for our batch size in order to maximize the likelihood of convergence. Since the learning rate is tightly connected with batch size, gradient accumulation and distributed training were used to maximize the batch size and accelerate pre-training. A few experiments were performed to determine the most optimal hyperparameters. Table 2 shows these parameters, along with the model configuration parameters. The optimal parameters selected are in line with the suggested hyperparameters given by Liu et al.^22^ Distributed training was performed using NCCL primitives with Fairseq 0.9.1 + Pytorch 1.7.1.^33^ A copy of the entire dataset was stored on each of the four GPU nodes, one of which was the master node. The dataset was split over the entire system, with each GPU containing a copy of the model. The optimized gradient accumulations from each node were then gathered using all-reduce,^34^ and the model’s parameters were updated.

**Table 2:** Model Configuration and Hyperparameters

hyperparameter	value
batch size per GPU (11 GB)	8
gradient accumulation steps	32
effective batch size	4096
peak learning rate	0.0006
hidden size	768
intermediate size	3072

### Pre-Training and Evaluation Pipeline

First, a new MFBERT model was initialized with random weights. The model was then pre-trained on the entirety of the shuffled GDB-13 dataset,^8^ and then training continued on the remainder of the aggregate dataset. For each part of the aggregate dataset, the model was trained for 1 epoch on 4 × 4 GTX1080Ti GPUs. Training on 1 epoch guarantees that all the molecules within the aggregate dataset have been seen by the model at least once. Further training may improve downstream performance; however, the returns on investment of additional pre-training time and compute diminish beyond 1 epoch. Pre-training on the entire dataset took approximately 3.5 weeks with this setup. Once the model was trained, the weights were transformed to be compatible with the HuggingFace’s transformer library^35^ for more accessible tokenization, inference, and fine-tuning environment. The MFBERT pre-trained checkpoint model was pre-trained for a further 0.5 epoch for use as a fingerprinting method in RDKit’s benchmarking platform for virtual screening.^23,24^ It also acted as a starting point for the fine-tuning procedure.

### GDB-13 Exploration

Given the size and diversity of the GDB-13 dataset,^8^ an ablation study was performed, where the model was independently trained against only the seven GDB-13 subsets^8^ to explore the impact of data diversity on the model’s bias and the impact each functional group may have on the results of the virtual screening task. For each subset, the model was trained using augmented datasets to account for the varying dataset sizes.

### Fine-Tuning Procedure

For fine-tuning, we took the mean of our pre-trained MFBERT model embeddings for each sample in the fine-tuning training set, added a 20% dropout on the mean of the embeddings, and then fed it through a single-layer feed-forward neural head with varying dimensions based on the number of classes within a given task. A sigmoid activation function was applied over the heads’ logits, and the binary cross entropy or mean squared error (MSE) loss functions were used for all classification and regression fine-tuning tasks, respectively. For inference, the neural head was removed such that the latent space containing the specialized molecular fingerprints could be accessed.

### Siamese-MFBERT

**Figure 4:**
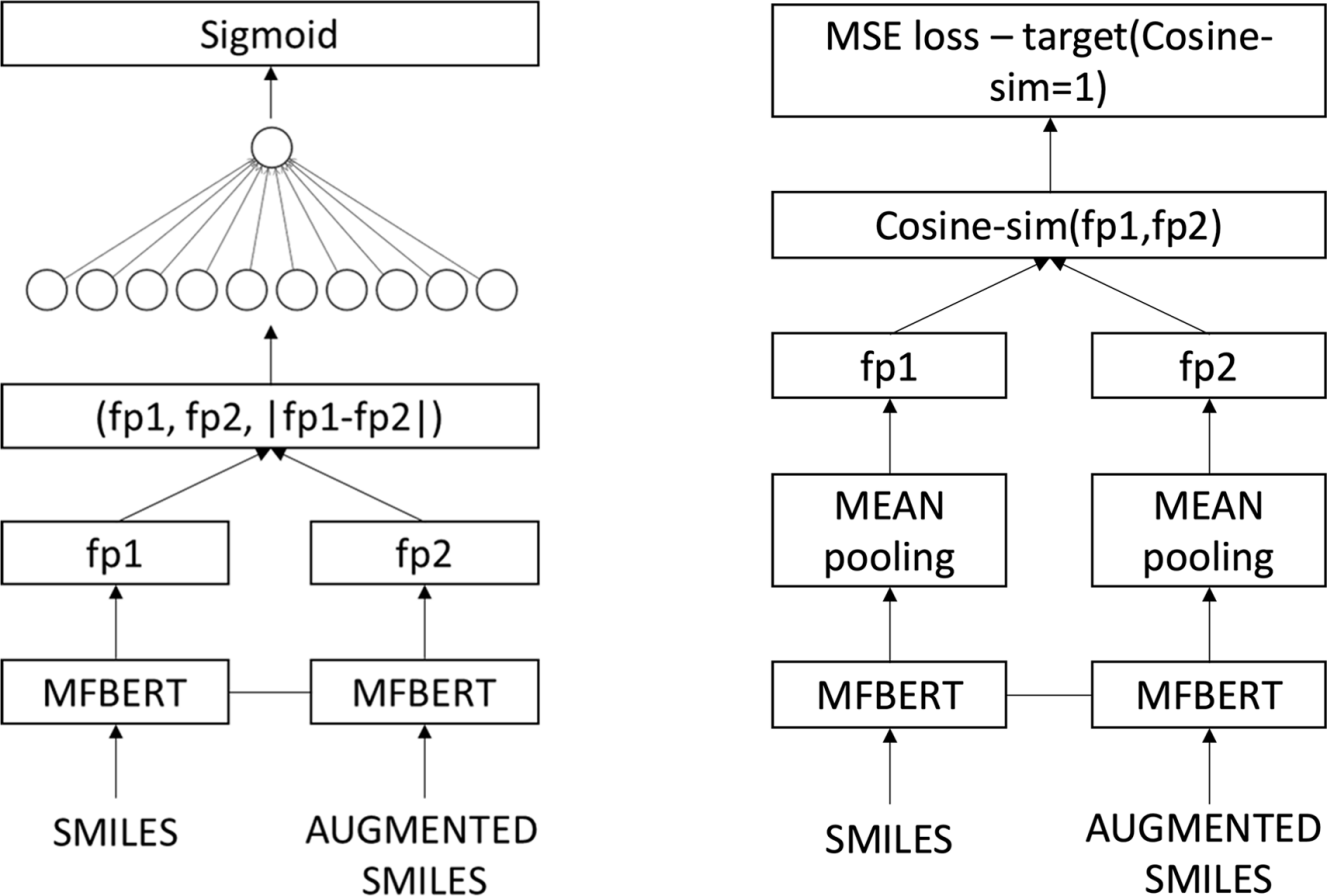
Siamese-MFBERT network architecture for both training strategies: classification (left) and augmented latent representations (right). The two MFBERT network weights are shared in both cases, providing the Siamese architecture.

Recently, works in NLP include taking advantage of multi-sentence inputs through Siamese BERT networks.^36^ We take inspiration from this work and devise two new training strategies with a Siamese-MFBERT network. These strategies open new avenues for transformers on augmented chemical data. These include (a) more elaborate uses of SMILES augmentation techniques (i.e., test-time augmentation with simultaneous inputs) and (b) new training tasks for learning more representative latent embeddings. Figure 4 shows an illustration of the two training procedures we devised for Siamese-MFBERT.

In both cases, we clone the already pre-trained MFBERT model as the starting point for the Siamese base. We fine-tune the classification network on three classification datasets from MoleculeNet^11^ and compare with the single MFBERT model on the same datasets. For each sample, during both training and inference, the SMILES is randomly augmented using RDKit’s traversal algorithm, and the augmented SMILES is fed with the original smiles to the model.

#### Siamese Classification

We concatenate the molecular fingerprints of both the original SMILES (fp1) and the augmented SMILES (fp2) and the element-wise difference between the two fingerprints, |fp2 – fp1|. This is then fed forward through a neural head of weights , where *n* is the dimensionality of MFBERT fingerprints (768) and *l* is the number of labels for the classification task. For binary classification with one label, we used a Sigmoid activation function, σ, with binary cross-entropy loss.
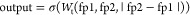


#### Augmented Latent Representations

For this task, we try to teach the model the similarities between a molecule’s SMILES and its augmented counterpart. Like the classification model, we compute both fingerprints. We then compute the cosine similarity between the two fingerprints and use MSE loss with a target cosine-similarity of 1 during training. This task ensures that in the latent space, every molecule and its various SMILES permutations are “similar” in cosine space.

## Results

RDKit’s benchmarking platform’s filtered subset (version 1.2)^24^ was used to evaluate the effectiveness of the generated molecular embeddings as a fingerprint for a virtual screening task. The dataset consists of 69 protein targets and pools of a small number of active molecules and many decoy molecules. The benchmark thus measures the performance of molecular representation (fingerprints) in separating active target molecules from decoys given a fixed number of query molecules (*n* = 5). The objective function used for compound retrieval is the cosine distance in the model embedding’s latent space. Standard retrieval metrics were used to enable comparison with other models on this task. The virtual screening metrics used were the (1) area under curve receiver operating characteristic (AUCROC) and (2) Boltzmann-enhanced discrimination of ROC (BEDROC) with ∝ = 20. The BEDROC20 metric is more discriminatory as it weights the top ∝% ranked retrievals higher in accordance with the Boltzmann distribution. This aims to aid with the problem of early recognition in which many of the virtually screened molecules do not make it into experimental testing because virtual screening databases are often too large.

### Inference Optimization

**Figure 5:**
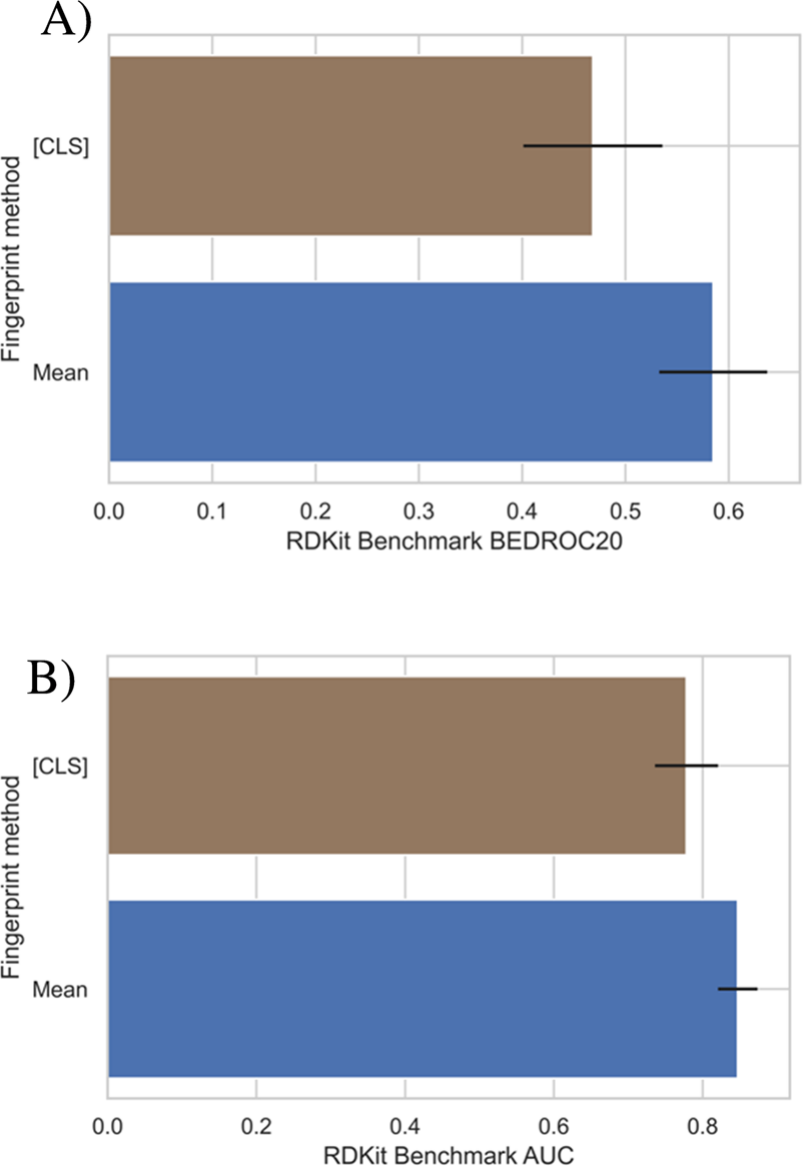
Comparison of the model’s performance on the virtual screening benchmark when using an aggregate token embedding and the mean of all token embeddings for the molecular fingerprint. (A) shows the BEDROC20 score, and (B) shows the AUCROC score for each inference method.

There are two paths in which inference for a molecular fingerprint could be performed: (1) using the [CLS] embedding as a token aggregator for all tokens in the molecule and (2) taking the mean of all token embeddings of the molecule. Figure 5 shows a comparison of the benchmark results between the two methods for the same model.

On both the AUCROC and BEDROC20 metrics applied to the virtual screening benchmark, taking the mean of the embedding as opposed to the aggregate [CLS] token drastically improves performance and reduces the uncertainty of the retrieval on this benchmark. One rationale for this improvement is that it is entirely dependent on the pre-training task (MLM). Since during training, each of the tokens was equally likely to get corrupted, the weighted importance for each token in each sample becomes approximately equal; this is useful for the SMILES representation since it is non-token-redundant. In contrast, this equal weighting becomes an issue in NLP as stop words are frequently present yet do not add any semantic value; as such, a token aggregator token is used. The [CLS] token is still included in our model as it provides greater flexibility for use in downstream tasks. When fine-tuning on a dataset for a specific task, our model’s [CLS] token can be used to generate a more suited fingerprint for each specific task; however, the model’s weights can also be frozen such that computational time and resources can be minimized for fine-tuning if needed, and the mean embedding molecular fingerprint can be used.

### Virtual Screening

**Figure 6:**
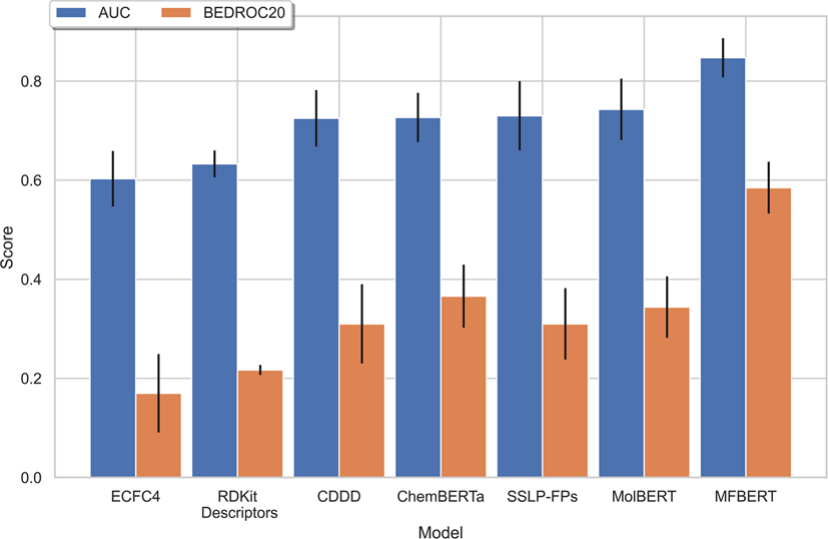
Comparison of MFBERT’s scores on the RDKit benchmarking platform with other cutting-edge fingerprinting methods from the literature.

We train MFBERT for a further 0.5 epoch on the aggregate dataset and compare it with five other molecular fingerprinting methods on the same benchmark, including the current state of the art. Figure 6 shows the results.

The fingerprinting methods we compare can be separated into two categories: data-driven methods and classical methods. We compare with (1) extended connectivity fingerprints (*d* = 4) (ECFC4), one of the most common molecular fingerprinting algorithms with common parameters; (2) RDKit descriptors,^28^ which fingerprints a molecule based on its physiochemical properties; (3) continuous and data-driven descriptors (CDDD),^1^ a deep learning-based encoder–decoder model for molecular descriptors; (4) ChemBERTa-^12^ a RoBERTa-based^22^ model designed for the prediction of molecular properties through transfer learning; (5) the self-supervised learning platform for molecular fingerprints (SSL-FP),^14^ a transformer encoder-based model trained on hundreds of millions of molecules; and 6) MolBERT,^13^ a BERT-based model for molecular representation and the current state-of-the-art for this benchmark.

MFBERT outperforms the current state-of-the-art method for this benchmark, with an average improvement of 15% in the retrieval score (AUCROC) and an improvement of 70% for the early recognition score (BEDROC20) over the next best model. For this benchmark, all data-driven methods for molecular fingerprinting outperform the classical methods that were tested. Our model maximizes this difference through training on the largest chemical dataset aggregate and utilizing an inference optimization technique more suited for general molecular fingerprints. We also fine-tune our Siamese-MFBERT model using the augmented latent representation strategy on a 1 million SMILES sample from our aggregate dataset and assess its performance on the benchmark. This strategy seems to generate inferior fingerprints for virtual screening with an AUCROC score of 0.554 ± 0.016 and a BEDROC20 score of 0.165 ± 0.015. This suggests that the model was limited to learning SMILES permutations rather than more accurate molecular representations.

### GDB-13 Exploration

There seems to be little correlation between data size and model performance for this benchmark. For example, the ChemBERTa model was trained on a much larger dataset than MolBERT, which suggests that there are other contributing factors/parameters within the training dataset that affects the model’s performance. Further evidence of this is given by Chen et al.’s work on SSLP-FPs.^14^ To explore the features that affect downstream performance, we train seven different models on the suggested cumulative GDB-13 subsets, each omitting some molecular features. We permute the SMILES before training such that all subsets are of the same size, and we adjust the learning parameters accordingly. Table 3 shows the GDB-13 subsets and the functional groups omitted from each set.

**Table 3:** GDB-13 Cumulative Subset Sizes and Molecule Removal Criteria

cumulated subset	subset size (molecules)	cumulative criteria
AB	635,647,478	no cyclic or acyclic HetHet bonds
ABC	441,084,370	stable FG
ABCD	277,628,675	no cyclic C=C or C≡C bonds
ABCDE	140,606,518	no acyclic C=C or C≡C bonds
ABCDEF	43,729,989	no small rings
ABCDEFG	12,899,741	fragment-like
ABCDEFGH	1,470,284	scaffold-like

**Figure 7:**
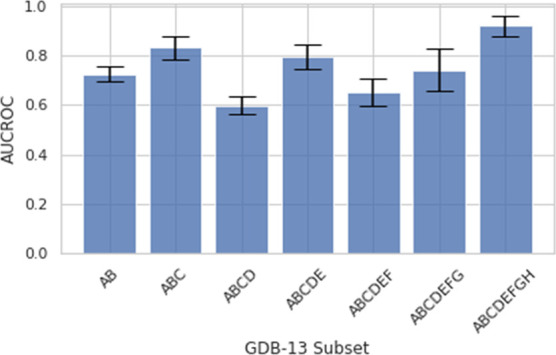
AUCROC scores with pre-training on a single GDB-13 subset.

For each subset, the model was trained with the same learning hyperparameters and evaluated on the same benchmark. Figure 7 shows the evaluation results for each set.

From these results, the cumulative ABCDEFGH subset achieved the highest score, closely followed by the ABC and ABCDE subsets. All other subsets performed slightly worse than these two subsets. This highlights how the convergence fragility of such large, stochastically optimized models can result in marginal gains or losses on a downstream task. This result also gives some insight for future training data pre-selection as to what causes the fluctuation. From previous works, we know that SMILES permutations do not tend to improve model performance for this task;^13^ thus, in this case, we can assume that our data augmentation to standardize the size of each of the subsets did not affect the results. The subset with the smallest initial size and the most general variations, scaffold (ABCDEFGH), performed the best on this virtual screening task. Since each of the models were trained for 1 epoch, the reduced initial number of molecules (and hence, the increased repetition through augmentation) allows the model to abstract the dataset more effectively, and the improved performance can therefore be explained. Increased data enrichment beyond scaffold-like molecules does not seem to improve performance given the constraints of the number of training epochs. Longer training may enable the model to abstract the additional features; however, in this particular benchmark, scaffold feature variations seem to be of utmost importance. This introduces the problem of generalization and highlights the importance of the quality of the data used for training as opposed to the quantity. We refer the reader to the GDB-13 paper^8^ for further details regarding the splits.

### Comparisons of Pre-Trained Fingerprints and Fine-Tuned Fingerprints

**Figure 8:**
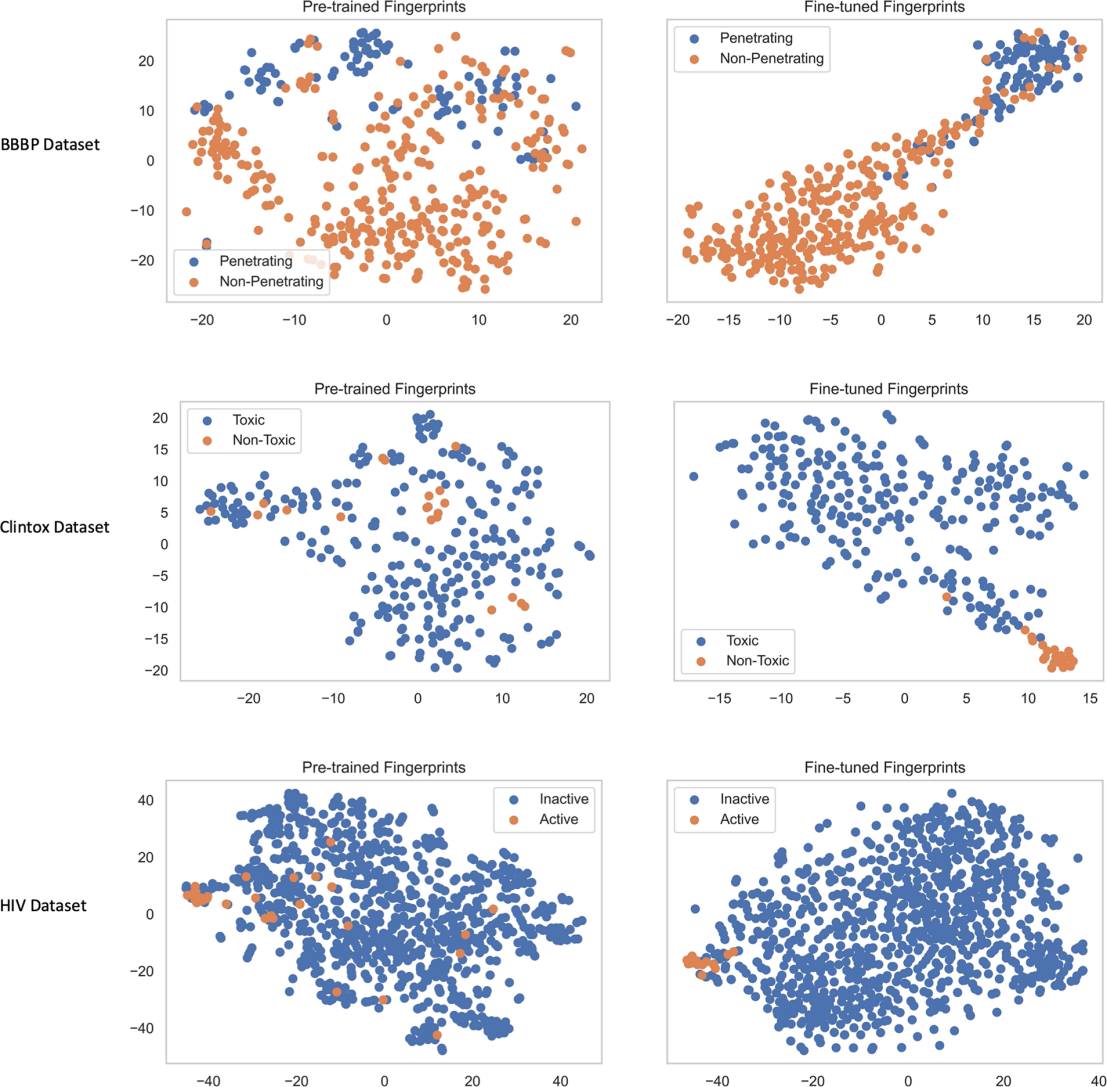
t-SNE visualizations for the molecular fingerprints generated by MFBERT before and after fine-tuning on the test sets of smaller targeted classification datasets.

We compare our model’s initial pre-trained fingerprints with the fingerprints generated after fine-tuning our model on three standard molecular classification datasets. These were the blood–brain barrier penetration (BBBP) dataset,^37^ ClinTox dataset,^38^ and the HIV dataset,^11^ all downloaded from MoleculeNet.^11^ During fine-tuning, the mean of the embeddings was used for training. For each dataset, the model was trained for 10 epochs with 80% of the data, with the other 20% used as test/validation sets; the split was done randomly for the t-SNE visualizations and K-means analysis. Figure 8 shows the t-SNE plots before and after fine-tuning on each of the three datasets. From the t-SNE plots, it is shown that the model’s pre-training allows for better performance on downstream tasks where only limited data is available. Even without fine-tuning, our model has learned a diverse representation of molecules. For example, without prior knowledge on either toxicity or blood–brain barrier penetration of molecules, our pre-trained model is able to somewhat separate molecules in the latent space that can penetrate the blood–brain barrier from those that cannot. This latent separation can also be observed across other tasks to varying degrees. Of course, the model’s performance is improved, and the fingerprints become more targeted after fine-tuning. We quantitively assess this difference for each task by performing principal component analysis (PCA) to reduce the dimensionality of the test-set fingerprints to 2D and then using K-means clustering to classify the data. Table 4 shows the AUCROC scores and the silhouette coefficients of the K-means clustering on the pre-trained and fine-tuned fingerprints.

**Table 4:** AUCROC and Silhouette Scores of our Fingerprints + PCA + K-Means for Three Classification Datasets^a^

	pre-trained fingerprint score		fine-tuned fingerprint score	
dataset	*	a	*	a
BBBP	0.589	0.416	0.741	0.671
ClinTox	0.533	0.471	0.814	0.722
HIV	0.529	0.364	0.744	0.679

a *AUCROC score; ^†^silhouette score.

### Comparisons of Performance on Downstream Tasks

Using our fine-tuning strategy and varying the model heads, we compare each possible combination of MFBERT fingerprint + head for both classification and regression tasks. For the regression datasets, we use (a) ESOL, a water solubility dataset; (b) FreeSolv, experimental hydration free energies in water; and (c) Lipophilicity, a dataset of experimental octanol/water distribution coefficients, all from MoleculeNet.^11^ For each dataset, we split the data into train/valid/test sets in 80%/10%/10% proportions, respectively; we follow the recommended splitting strategy for each dataset as described in MoleculeNet.^11^ For each regression task, we use a random splitting strategy. For the classification datasets (with the exception of ClinTox, where we also do random splitting as recommended), scaffold splitting from DeepChem^39^ was used. For the regression tasks, we take both fine-tuned and pre-trained MFBERT fingerprints with a (1) support vector machine (SVM) regressor, (2) random forest (RF) regressor, and (3) feed-forward neural network (FFNN). These heads were implemented with Sci-Kit Learn.^40^ We take the classification counterparts of these heads and apply them to the classification datasets. We also use our Siamese-MFBERT classification model on the three classification datasets. Table 5 shows the results of the various heads on each of the tasks. We then compare our results with other models in the literature applied to the MoleculeNet benchmark, including SSLP-FPs,^14^ ChemBERTa,^12^ MolBERT,^13^ and the best-performing model from the MoleculeNet results database.^11^Table 6 shows these results.

**Table 5:** Comparison of the Performance of Different Model Heads on Both MFBERT and Siamese-MFBERT across Six Datasets from MoleculeNet^a^

	MFBERT						Siamese MFBERT		
dataset model head	ESOL^a^	Freesolv^a^	Lipophilicity^a^	BBBP*	ClinTox*	HIV*	BBBP*	ClinTox*	HIV*
raw MFBERT + SVM/SVR	1.169 ± 0.161	3.021 ± 0.525	0.874 ± 0.047	0.661 ± 0.037	0.500 ± 0.000	0.500 ± 0.000			
raw MFBERT + RF	1.287 ± 0.146	2.515 ± 0.360	0.999 ± 0.061	0.759 ± 0.042	0.555 ± 0.009	0.532 ± 0.016			
fine-tuned MFBERT + FFNN	1.153 ± 0.141	1.180 ± 0.440	0.868 ± 0.021	0.762 ± 0.000	0.912 ± 0.000	0.765 ± 0.000	0.750 ± 0.000	0.913 ± 0.000	0.765 ± 0.000
fine-tuned MFBERT + SVM/SVR	0.423 ± 0.501	0.999 ± 0.450	0.366 ± 0.032	0.689 ± 0.048	0.864 ± 0.099	0.511 ± 0.009	0.686 ± 0.062	0.867 ± 0.092	0.511 ± 0.013
fine-tuned MFBERT + RF	0.600 ± 0.040	0.624 ± 0.130	0.461 ± 0.032	0.726 ± 0.048	0.837 ± 0.186	0.612 ± 0.019	0.724 ± 0.043	0.845 ± 0.033	0.612 ± 0.064

a *AUCROC score; ^†^root mean squared error (RMSE) score.

**Table 6:** Comparison of the Performance of MFBERT with Other State-of-the-Art Models from the Literature^a^

	MFBERT (+FFNN)	MFBERT (best)	ChemBERTa	SSLP-FPs (best)	MoleculeNet (best)	MolBERT (fine-tune)
BBBP*	0.762	0.762	0.663	0.741	0.729	0.762
ClinTox*	0.912	0.912	0.842	0.963 ± 0.044	0.907	0.912
HIV*	0.765	0.765	0.643	0.601	0.792	0.783
ESOL^†^	1.153 ± 0.141	0.423 ± 0.501		0.915 ± 0.01	0.58	0.531 ± 0.04
FreeSolv^†^	1.180 ± 0.440	0.624 ± 0.130		0.891 ± 0.075	1.15	0.948 ± 0.33
lipophilicity^†^	0.868 ± 0.021	0.366 ± 0.032		0.717 ± 0.029	0.655	0.561 ± 0.03

a *AUCROC score; ^†^root mean squared error (RMSE) score.

All models were fine-tuned and tested on the same splits for consistency in this experiment. For the ChemBERTa model, MFBERT’s mean strategy was applied to the contextual embeddings followed by an SVM classifier for an improved score and better comparability. For the MoleculeNet (best) scores, different models were used for each task. For the BBBP and HIV tasks, the scores were achieved using RDKit’s ECFP4 + KernelSVM; the ClinTox score was achieved using a weave molecular model, the ESOL and FreeSolv scores were achieved with a message-passing neural network, and finally, the Lipophilicity score was achieved using a graph convolution model. These models’ scores were taken from the MoleculeNet benchmark results.^11^ MFBERT’s approach uses a single pre-trained architecture, which can be fine-tuned to perform many different tasks using a single pipeline. While the following transformer-based models (MFBERT, SSLP,^14^ ChemBERTa,^12^ and MolBERT^13^) are similar architecturally, the differences in scale, training data, pre-training procedure, and inference optimizations have shown to significantly affect downstream performance. For the classification tasks, there seems to be no correlation with the model’s pre-training data size and downstream performance; for the regression tasks, models with larger pre-training data sizes consistently outperformed those with smaller pre-training datasets.

The AUCROC scores match the t-SNE plots in terms of class separation. For the BBBP dataset, our model achieves an AUCROC score of over 0.58 with no knowledge or training of the task. For the BBBP task, since there is a correlation between molecular size and BBBP, it seems that during pre-training, the model has learned some features regarding molecular size. This suggests that the patterns recognized by the model remain constant for a variety of molecules and that the feature representation is valid. The silhouette score further confirms that while the pre-trained fingerprints offer some separation between clusters across the downstream tasks, this separation is massively enhanced with fine-tuning.

## Conclusions

In this work, we have trained one of the largest deep learning models in the chemical literature for generating molecular fingerprints on a custom-prepared aggregate dataset of over 1.2 billion molecules. We used the power of distributed computing to perform such large-scale training in a reasonable timeframe of 1.5 months. We evaluate our model on RDKit’s benchmarking platform and compare its performance with other models on the same virtual screening task. We found that taking the mean of the output embeddings significantly outperforms using an aggregate [CLS] token for generating molecular fingerprints for virtual screening. Utilizing this as an inference strategy, our model achieves state-of-the-art performance on the virtual screening benchmark with over 70% improvement in the BEDROC20 score over the next best model. We also explore the impact of discriminating the molecular variety from the GDB-13 dataset^8^ on the model’s performance. This highlighted the importance of the quality of pre-training data on the generated molecular fingerprints while also emphasizing the fragility of training such large models and that a larger data-set size does not necessarily mean better downstream performance. We introduce the process of fine-tuning MFBERT and its Siamese variants for augmented SMILES inputs to generate more targeted molecular fingerprints. We also train a selection of classical ML heads on top of MFBERT fine-tuned fingerprints and compare the performance with other models in literature. We explore the separation of the classes in latent space with techniques such as PCA and K-means to perform classification using the pre-trained and fine-tuned fingerprints. We leave to future work further exploration of fine-tuning with ablation studies on the inference method, model weight freezing, and performance of predicting more niche properties from the fingerprints.

## Notes

The datasets that were used for training the model are all publicly available and can be accessed through the following references: GDB-13,^8^ Zinc 15,^25^ PubChem,^26^ ChEMBL,^27^ and USPTO.^9^ All of the model weights determined and presented in this work are available on figshare (https://figshare.com/articles/software/MFBERT_Model_weights/15048381). The training and inference code developed for this article is available from https://github.com/GouldGroup/MFBERT .

## ABBREVIATIONS

MFBERT: molecular fingerprints through bidirectional encoder representations from transformers
AUCROC: area under curve receiver operating characteristic
BEDROC: Boltzmann-enhanced discrimination of ROC
BBBP: blood–brain barrier penetration
